# New frontiers in the future of palliative care: real-world bioethical dilemmas and axiology of clinical practice

**DOI:** 10.1186/s12910-015-0003-2

**Published:** 2015-02-26

**Authors:** Uría Guevara-López, Myriam M Altamirano-Bustamante, Carlos Viesca-Treviño

**Affiliations:** Facultad de Medicina y Cirugía de la Universidad Autónoma Benito Juárez de Oaxaca, Oaxaca, Mexico; Centro Interdisciplinario para el Estudio y Tratamiento del Dolor y Cuidados Paliativos del UMAE “Dr. Victorio de la Fuente Narvaez”, México, DF Mexico; Grupo Transfuncional en Ética Clínica, Centro Médico Nacional Siglo XXI, IMSS, México, DF Mexico; Unidad de Investigación de Enfermedades Metabólicas, Centro Médico Nacional Siglo XXI, IMSS, México, DF Mexico; Facultad de Medicina, UNAM, México, DF Mexico

**Keywords:** Palliative care, Bioethical dilemmas in real life, End of life, Axiology, Values and virtues

## Abstract

**Background:**

In our time there is growing interest in developing a systematic approach to oncologic patients and end-of-life care. An important goal within this domain is to identify the values and ethical norms that guide physicians’ decisions and their recourse to technological aids to preserve life. Though crucial, this objective is not easy to achieve.

The purpose of this study is to evaluate empirically the real-life bioethical dilemmas with which palliative physicians are confronted when treating terminal cancer patients.

**Methods:**

A quasi-experimental, observational, comparative, prospective and mixed (qualitative and quantitative) study was conducted in order to analyse the correlation between the palliative doctor-patient relationship and ethical judgments regarding everyday bioethical dilemmas that arise in palliative clinical practice. The values at stake in decision-making on a daily basis were also explored.

From February 2012 to march 2014, palliative healthcare personnel were invited to participate in a research project on axiology of clinical practice in palliative medicine. Each participant answered to a set of survey instruments focusing on ethical dilemmas, views, and representations of clinical practice.

For this analysis we selected a convenience sample of 30 physicians specialized in pain medicine and palliative care (algologists and palliativists), with two or more years of experience with oncologic patients and end-of-life care.

**Results:**

113 dilemmas were obtained, the most frequent of which were those regarding sedation, home administration of opioids, and institutional regulations. We observed that the ethical nucleus of palliative medicine is truth-telling, implying bidirectional trust between patients and healthcare providers. The two most prominent virtues among the participants in our study were justice and professional humility. The outstanding roles of the physician in palliative medicine are as educator and as adviser, followed by that of provider of medical assistance.

**Conclusions:**

This investigation opens up new horizons in a career path where professional wearing is rampant. The rediscovery of values and virtues in palliative clinical practice will renew and replenish the motivation of healthcare providers who carry out these duties, giving them a new professional and personal perspective of growth.

## Background

Cancer is a class of diseases involving an uncontrolled production of cells resulting from changes in the genetic information of the cells themselves. In human beings, cancer is a complex process which has an impact on several areas of the life of individuals, families, and society at large. In terms of global public health, cancer is the leading cause of death in developed countries and the second cause of death in developing countries. In 2008 there were 12.7 million cases of cancer, causing 7.6 million of deaths worldwide, and it is expected that by the year 2020, 15 million new cases per year will occur [1,2]. Beyond statistics, cancer creates immeasurable distress to patients and their families, not only from a purely biological perspective, but also in their psycho-social stability and well-being.

Over the past few decades interest in a systematic approach to end-of-life care in oncologic patients has been growing. A crucial objective in this respect is identifying the values and ethical norms that guide the physician’s acting, and his or her recourse to technological aid for preserving life. Evidently, this is not an easy task.

The focus of this article is studying the most salient ethical dilemmas that arise during the stage of palliative care in oncologic patients. The key questions we hope to answer are: 1) what are the main real-world bioethical dilemmas that emerge when providing palliative care for terminal patients? 2) How do palliative care physicians solve real-world bioethical dilemmas in clinical practice? 3) What core values guide the decision-making processes of palliativists? These are open questions, and their answers have fundamental implications for our conception of palliative medicine and its values.

The global objectives of this research were:

- To perform a cross-functional analysis of the ethical dilemmas that palliative physicians encounter in clinical practice.
- To make an ethical discernment of issues regarding the care of patients at the end of their life.

Specific objectives:

1. To identify the most common ethical dilemmas in palliative medicine in México.
2. To perform an axiological analysis of the narratives of the dilemmas that palliative physicians face.
3. To make a systematic inquiry of how ethical discernment of dilemmas in this medical specialty are made.
4. To analyse the ethical discernment of dilemmas particular to this specialty from an axiological perspective.

In this work, we define a dilemma as an issue when two courses of action are possible, each favoring certain values over others, producing different results. Very often, however, the specific factors intervening in these options are difficult to assess, rendering decision making very complex. In order to strengthen the evidence-based: values-based medicine binomial, it is necessary to develop a deeper awareness of what it means to be a physician as well as the goals of medicine, in order to cultivate virtues of moral reasoning and moral attitudes. In other words, to foster an environment in which healthcare personnel are ever more aware of their decisions, becoming more responsible about their actions in daily clinical practice.

Ethical dilemmas can mainly be viewed from three different moral standpoints, which are sometimes in conflict with one another: emphasizing the effects of the action, which can be utilitarian, consequential or pragmatic; emphasizing the nature or the process of the act itself, which we call deontological or procedural; and those emphasizing the realization of the virtues of the moral agent as such, which are aretological or of virtue [3]. The utilitarian approach is innate and thus the most straightforward of the three, proposing that an action is good to the extent that its effects, effectiveness or efficiency are good: something is good if I can observe, measure and perceive it, and if there are effects or tangible consequences of its performance. In terms of the deontological perspective all forms of duties and rights to be kept are considered, so that the professional service is properly given and does not become merely an instrument to meet ones needs, preferences, likes or virtues, but rather works towards the common good and the fulfillment of a function within a social or natural order. The aretological standpoint (which comes from the Greek ‘areté’, meaning virtue) centers on the agent and the principal aim of all human action that is human beings. From this view, an action is good when the moral agent acts freely, acquires virtues, flourishes, and reaches his fullness [3].

In the present work we developed a clear and rigorous empirical study of the axiology^a^ of clinical practice in palliative medicine, in which we explain how different the tasks of a palliative physician are in relation to clinical practice in other medical specialties. In palliative medicine, the autonomy and the right of a sick person to choose, reject or interrupt treatment are frequently ignored. A number of factors such as: severity of disease, cognitive disorders, neurologic injury, and sedation hinder non-curable patients diagnosed as palliative to participate in medical decision-making regarding measures to provide comfort, anxiolysis or treatment for associated symptoms. In such situations, patients can be medically unable to exercise their autonomy and it is the family or the therapeutic team who take the decisions on their behalf [4-7].

Palliative physicians constantly face dilemmas such as whether they should rely on subrogated decisions or not; whether to apply interventional procedures or not; whether to administer controversial measures like palliative sedation; whether to continue or stop life supporting procedures like dialysis, hydration/nutrition, anti-infectious medicine, hypercalcemia therapy, opioid painkillers or analgesics, blood transfusion, or whether to prolong life by means of aggressive methods in cases when there is short life expectancy [8,9].

Medical attitudes towards such cases vary widely, given that there is not a model of behavior considered to be correct or infallible. Frequently, it depends on the ethical principles and values of individual physicians and individual cases [8,9]. An important challenge, then, is to approach practicing palliative physicians in high-specialty hospitals by means of ethnographic-anthropological reconstruction, so they can learn more about the values applied in their day-to-day work by having a clearer picture of the correlation between aspects such as educational background, life story, and ethical discernment in the physician-patient relationship. This study is a methodological qualitative and quantitative cross-functional approach to the contemporary palliative medical practice.

## Methods

### Study design

**Figure 1:**
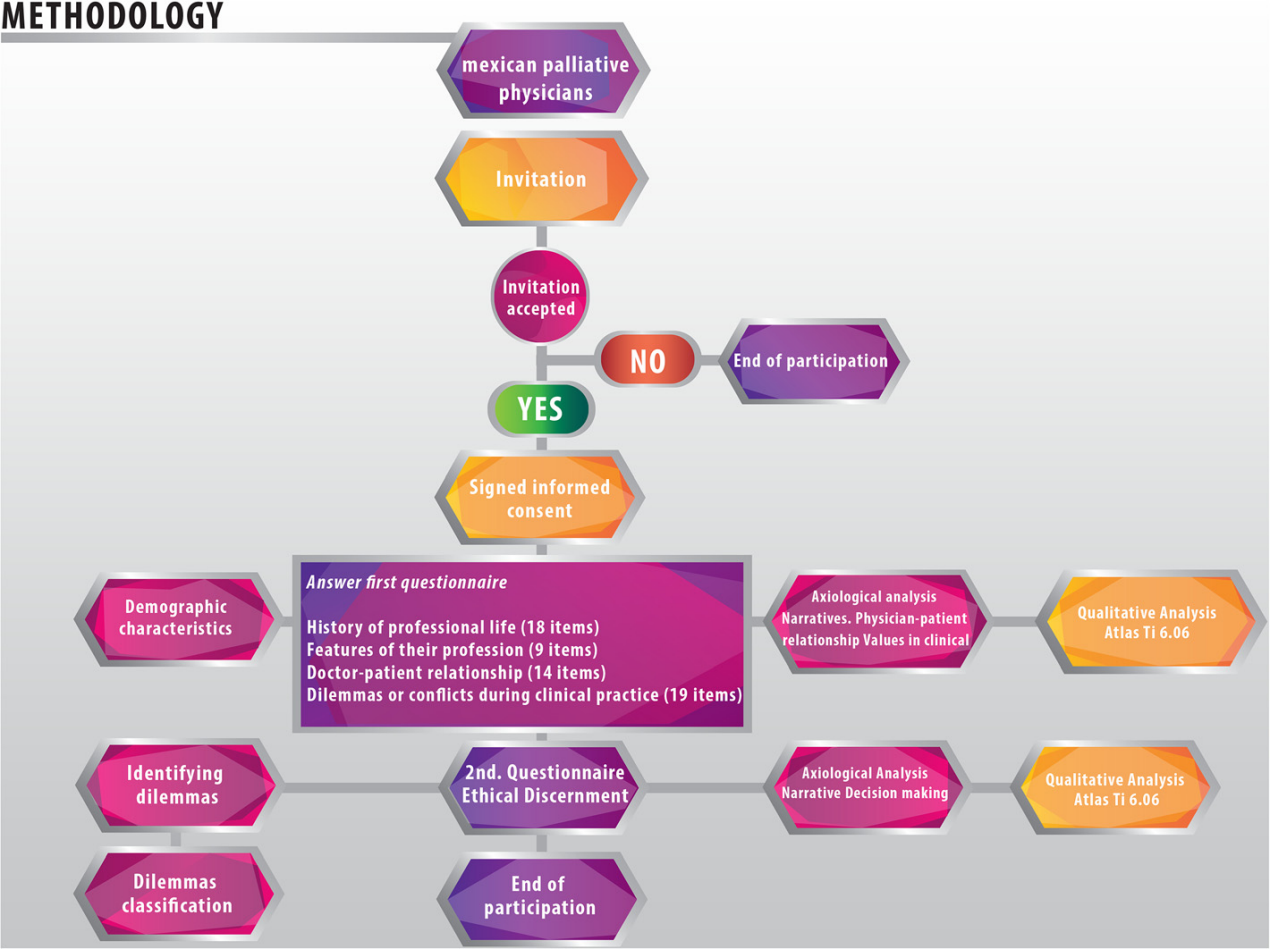
**Framework of the methodology and participating palliative physicians.** All palliativists from Mexico were invited to participate in this study. Of these, 30 accepted the invitation and signed their informed consent. In the initial stage physicians answered the first questionnaire (structured survey), which provides the demographic information of the participants and examines the values of the clinical practice, life history and the doctor-patient relationship, among other things. The open answers of the participants in this survey were codified to carry out the axiological analysis. The results of this analysis are shown in Figure 3. In the second stage, the palliative physicians were asked to narrate an ethical dilemma and to analyse it using the integral method. At this point, the participation of the palliative physicians ended. The narrations were codified in order to identify the most prominent values and virtues at work (Figures 3 and 5). A bank of dilemmas was created.

A descriptive, observational, prospective and mixed study (qualitative and quantitative) was conducted to identify the characteristics, frequency and nature of the dilemmas presented to specialist physicians in palliative care who take care of cancer patients diagnosed as not curable and/or cancer patients at the end of their life. It also sets out to determine the values and other factors that intervene in ethical discernment in decision-making in these processes Figure 1.

### Universe of study

A sample of 30 medical specialists in pain medicine and palliative care was studied (Algogists-palliativist) with two or more years of experience in caring for cancer patients and end of life at the “Centro Interdisciplinario para el Estudio y Tratamiento del Dolor y Cuidados Paliativos de la Unidad Médica de Alta Especialidad ‘Dr. Victorio de la Fuente Narváez’ DF” (Interdisciplinary Center for the Study and Treatment of Pain and Palliative Care of the Medical Unit of High Specialty ‘Dr. Victorio de la Fuente Narváez’ DF), after obtaining the approval R 2012-785-006 of the National Research Committee of the IMSS (Mexican Institute of Social Security).

After obtaining informed consent of the interviewed physicians who were surveyed, a questionnaire designed and validated by a cross-functional panel was applied. The main aim was to identify the ethical dilemmas that palliativist physicians face when caring for cancer patients in the period between February 2012 and March 2014 Figure 1.

### Measurement instruments

#### Structured survey

The first questionnaire used was a structured survey (Figure 1), which allowed us to explore the experiences and representations that the participants of this study have about their own practice. This allowed us to describe the state of the art regarding axiology in palliative medicine.

The survey, based on the instrument designed by Altamirano and Cols [10,11], adapted to palliative care and validated by a cross-functional panel of experts, was given to the participating physicians who answered anonymously. In a nutshell, the structured survey covers: a) History of professional life (18 items), b) characteristics of their profession (9 items), c) Physician-Patient Relationship (14 items), d) Dilemmas or conflicts during clinical practice (19 items), d) Medical ethics (12 items) and e) Final impressions (3 items).

#### Ethical discernment

A second questionnaire was provided in the next step of participation (Figure 1), which included the comprehensive method of ethical discernment as a guide, in which a dilemma was holistically analysed taking the main bioethical strands into account: deontology, virtue ethics, and utilitarian vision [9]. This comprehensive method allowed us to identify the values at stake in decision-making of the reported dilemmas.

This method arises from the theoretical expectancy of fostering a clinical practice with high ethical standards in harmony with the available biomedical techno-scientific development, which considers the three aspects of clinical activity: moral agent, action (rights and obligations) and consequences of the action, Thus, it is ensured that for such discernment, different strands determined by the empirical circumstances are considered, with objectivity and flexibility, based on ethical principles which improve the relationship of the health team with the end-of-life patient.

#### Qualitative study

The qualitative side of this study seeks to explore the self-representations of health professionals when dealing with oncological patients at their end-of-life. The structured survey was given to participants to answer anonymously. It sought to identify their perspectives and representations. A total of 30 structured surveys were conducted. The surveys were transcribed and analysed using the method suggested by De Hoyos et al. [11], which consists of the familiarization with the data to subsequently establishing a conceptual framework which allows establishing codes that can be used to analyse the interviews and their further interpretation. The axiological framework on the basis of Schwartz’s work on values [12], the Oakley & Cocking’s studies on professional virtues [13], as well as the cultural competencies of clinical practice of Campinha-Bacote [14] served as basis for the design of this intervention.

The main issues emerging from our analysis of the interviews were the beliefs, wishes, meanings and their interactions, and how clinical practice is structured from the perspective of a given set of values. Each interview sought to achieve qualitative understanding of perceptions, attitudes and moral values relevant to this study [15]. Atlas ti 6.0 software was used to codify emergent issues and concepts in the participants’ narratives, in preparation for their analysis. One hundred and ten codes were established in the following categories: life history, ethical discernment, physician-patient relationship, cultural competency, decision-making, ethics committees and future expectations of health personnel.

## Results

**Figure 2:**
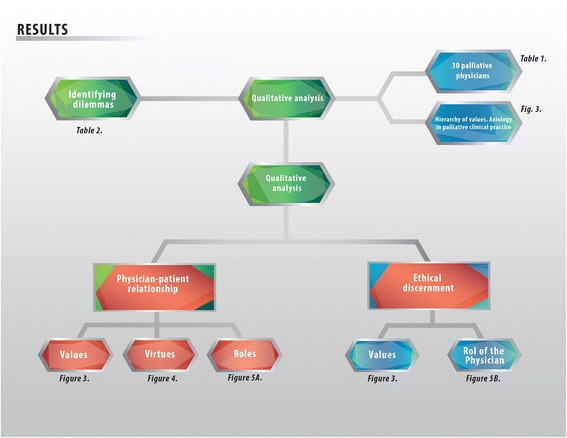
**Flow diagram of the results obtained in the study.** The emergent issues, namely, values hierarchically ordered, the doctor-patient relationship, and the ethical discernment of palliative medicine are shown.

As illustrated in Figure 2, regarding the demographic characteristics of the doctors interviewed it was found that they have: an average experience of 6.7 years, ranging between 2 and 28 years. Among them, 18 were women and 12 men, with an average age for women of 32 and 38 for men. 16 of these palliativist doctors are working in public institutions, and 14 in private institutions (Table 1).

**Table 1:** **Demographic characteristics of the physicians specialized in palliative care**

**Gender**	**Number of participants**	**Average age (years)**	**Average professional experience (years)**	**Institutional clinical practice**	**Private practice**
**Female**	18	32	2.7	11	7
**Male**	12	38	7.5	5	7
**Total**	30			16	14

N = 30.

When talking about dilemmas, these physicians often referred their experiences with various diseases including breast cancer, prostate cancer, osteosarcomas, among others (Table 2).

**Table 2:** **Real-world bioethical dilemmas reported**

**Cause of the dilemma**	**No.**	**%**
Sedation	13	11.5
Institutional regulations	11	9.7
Administration of opiates at home	11	9.7
Information of the diagnosis	10	8.8
Double effect	10	8.8
Communication of the diagnosis	9	8
Administration or removal of electrolytes	8	7
Hydration	8	7
Request for euthanasia	5	4.4
Removal of the food	5	4.4
Futility	5	4.4
Cultural and religious	4	3.5
Intergroup conflict	4	3.5
Blood transfusion	3	2.6
Concealment of the family	3	2.6
Suicide attempt	2	1.8
Administration of antibiotics	2	1.8
Total	113	100%

### Main dilemmas of palliative medicine

From the 30 interviews to palliativist doctors, 113 dilemmas of were obtained and classified in 17 categories according to their cause. In order of frequency, the most prominent ones were: sedation, institutional rules, home opioids, information regarding prognosis, double effect, communication of the diagnosis, management or withdrawal of hydration, request for euthanasia, withdrawal of food, futility, cultural and religious issues, intergroup conflicts, blood transfusion, the family request of no information to patients, attempted suicide and antibiotics (Table 2). This confirms the findings described in the literature [5,7,15-17]. A remarkable finding of our survey, however, is the frequency and relevance of dilemmas related to diagnosis and prognosis, which evidences the relevance of physician-patient relationships as the core of clinical practice in palliative care.

### Axiological analysis of ethical dilemmas in palliative medicine

Axiology is a philosophical discipline which studies values and phenomena around them. The values are normative systems, which allow us to understand whether an object or situation is good, appealing or desirable towards certain aims [10]. These ends and the values related to them give shape and meaning to human life on a daily basis.

**Figure 3:**
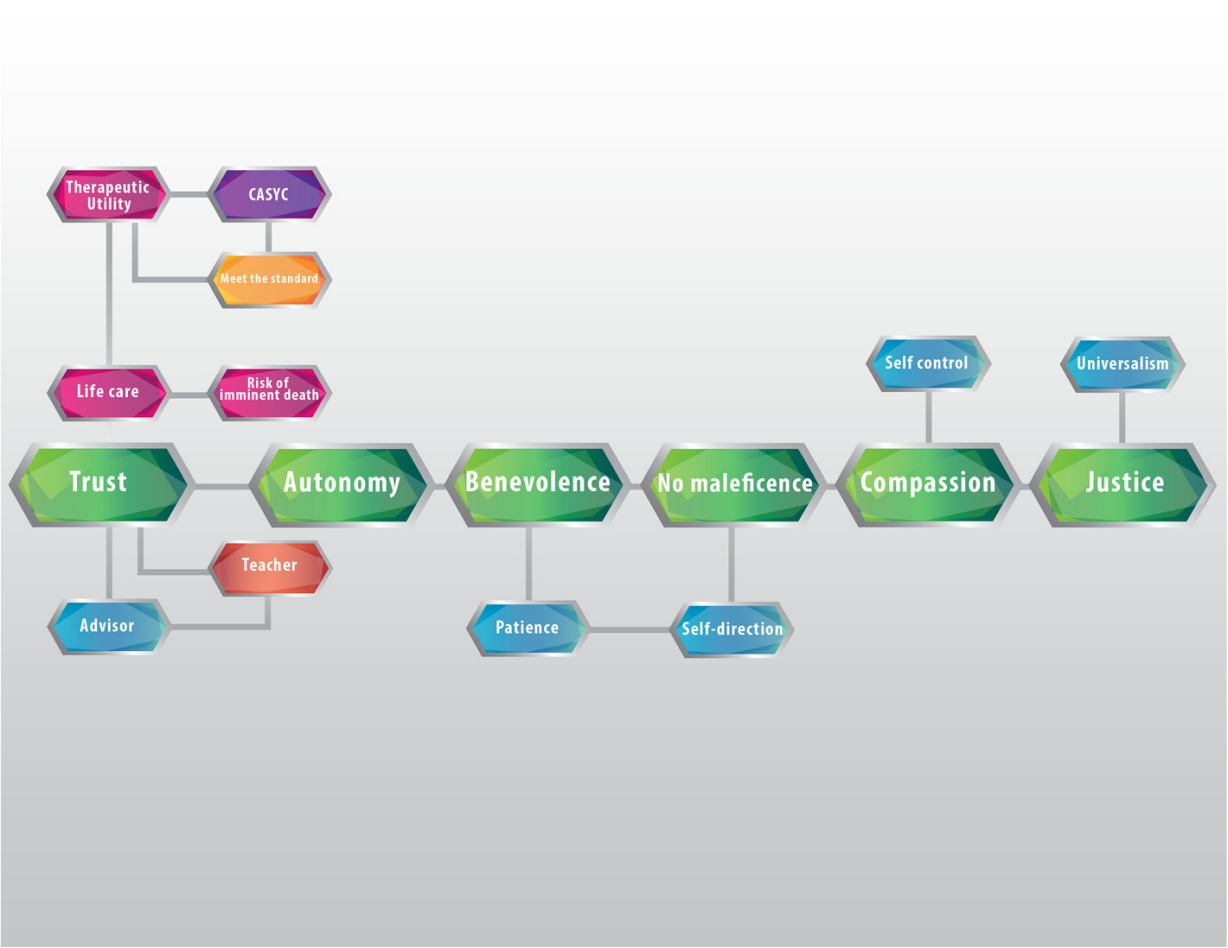
**Values analysis of ethical dilemmas in palliative medicine.** Values are normative guidelines that allow us to consider actions, objects or situations as good, desirable, pleasant, convenient or useful, in this case towards the fulfillment of ends of medicine (healing, curing and caring). The core of values of palliative medicine, encompass compassion, non-maleficence, benevolence, autonomy, and confidence. Trust helps build two virtuous circles: first, the palliativist’s role as an educator and counselor, and secondly, care of life, which involves a risk of imminent death, and therapeutic utility.

This is a pioneer study in terms of the empirical exploration of the values of healthcare professionals in palliative medicine in Mexico (Figure 3). Our study reveals the values specifically related to daily clinical practice regarding positive attitudes and behaviours that enable an effective clinician-patient relationship centered on the well-being of the former. The axiological backbone is made up of values such as trust, autonomy, and beneficence, non-maleficence, compassion, justice, and respect. The main role of trust as a predominant value in the qualitative analysis means that the ethical nucleus of palliative medicine is loyalty to the truth, expressed in a bidirectional trust, between a patient and healthcare provider, which creates an encounter between two people.

As Pellegrino used to say “without truth there is no future and actions stop” [18]. Trustworthiness is vital in the clinical practice, but it becomes sine qua non in critical moments of human interaction, as is the case of palliative care. Telling the truth generates confidence, which is an ineluctable element in human relations, and has strengthened medical professionals since the beginning of medicine. It enables patients to act, and to predict the future.

As soon as someone decides that he or she needs help, this person becomes a patient (suffering from anxiety, pain, and sorrow). From the very moment a patient seeks professional help, he or she commits an act of trust. They trust the doctor’s ability to help and heal them. The doctor, in return, trusts that the patient is telling the truth, which will help to produce a diagnosis and a plan of treatment. The results of our study show that trust has as a concurrent value “care for life”, which feeds on values such as “therapeutic utility”, “the ability to assess situations and their consequences”, as well as “meeting the current norms”. These values provide structure for many of the practices and decisions taken when there is an imminent risk of death (Figure 3).

In the configuration of the ethical nucleus of palliative medicine, a set of three values plays an essential role: autonomy, beneficence, non-maleficence (Figure 3). This triplet continues to be the guide between doctor-patient relationships until the end of life, because physicians seek the well-being of the patient. To foster the latter it must be considered: a) what is medically suitable from the physiological point of view to preserve physical and mental homeostasis; b) what is good for the patient according to his or her own perspective of what is good for him or her; c) what is good for him or her as a member of the human species; d) that which is good for the person from the spiritual point of view [18-21].

Acknowledging the importance of this set of three values allows physicians to become protectors and facilitators of the self-determination of patients and their network of supporters. This implies a joint responsibility in the therapy alliance, to refine, regulate, and discern proportionate from disproportionate measures, in order to avoid therapeutic cruelty.

These three values work in tandem with compassion and justice, which makes up together the axiological backbone of these situations (Figure 3). Compassion means “to suffer with” or “to suffering together”. Good physicians are aware of the suffering, the pain, and the patient’s loneliness. They share this suffering to an extent, knowing the dimensions and of the reach of human pain. In ideal circumstances, this fosters actions based on love, and on the desire to ease the pain of the person in front of them.

On the other hand, justice focused on abilities is in keeping both with institutional guidelines and the role of individual doctors and it also allows for deeper immersion in the wishes and interests of patients, their social networks of support. It should be remembered that justice is only possible when reasoned and iterated as a dialogue among interested parties [22].

When exploring the values particular to doctor-patient relationships in palliative medicine, compassion and professional humility come first, followed by trust. These three values play a leading role among others, such as the search for knowledge and self-control (Figure 3).

### The virtues in palliative medicine

According to Aristotle, “virtue makes good those who possess it, in other words, it perfects them”. Following this, Macintyre proposes that, “a virtue is an acquired human quality, whose possession is an exercise that enables us to achieve those goods, which are internal to the practices and whose lack hinders us from effectively achieve any of such goods”.

**Figure 4:**
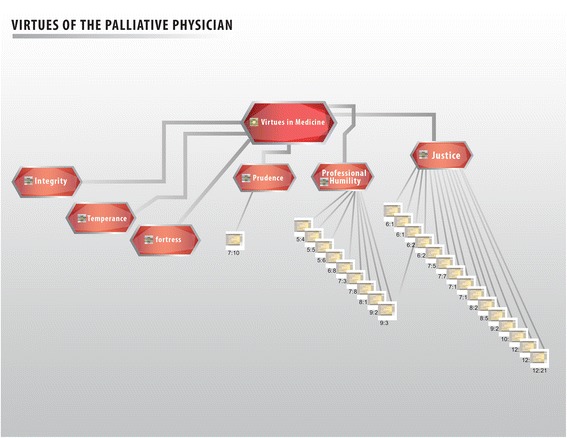
**Virtues of healthcare personnel.** Virtues are the values that refer directly to the healthcare personnel, their traits of character and decision-making. The main virtues observed were security, temperance, strength, prudence, professional humility, and justice.

The virtues of palliative medicine health care professionals are shown in Figure 4. The most prominent virtues in our group of study were justice and professional humility. Justice requires that each person gets what they need (therapy alliance), and that alike cases receive the same attention. Healthcare professionals must adjust themselves to the specific needs of the sick person. Professional humility, on the other hand, implies pacing the patient and his needs first. Humility allows us to recognize human excellence, becoming aware of our own imperfection. The virtue of humility allows us to recognize our limitations and those of others. In the case of the medical profession, it prevents physicians from taking advantage of the patients’ vulnerability by acknowledging them as persons. Humility reminds physicians of the ultimate purpose of their profession; it keeps them grounded on reality, fostering objective behaviours that allow them to concentrate on the implications of their decisions and actions, which have an impact in the patient welfare.

Prudence, strength, and temperance are other important virtues that medical personnel working in palliative care claimed to follow in their daily practice. Prudence can be defined as “knowledge of the good and the disposition or applying it in special circumstances”. Plainly speaking, it can be said to be the “correct form of acting” [18,22]^.^ It helps us to discern and to make the best decision (so important in ethical dilemmas), particularly in times of lack of clarity, or when there is a counterpoint. It acts as a sort of lighthouse for other virtues, guiding them to safe port in the middle of the ocean.

### Roles of the doctor in palliative medicine

Lain Entralgo [23] defines the patient-physician relationship as a quasi-dyadic relationship, whose structure is formed by

1. advice
2. education
3. medical attention

**Figure 5:**
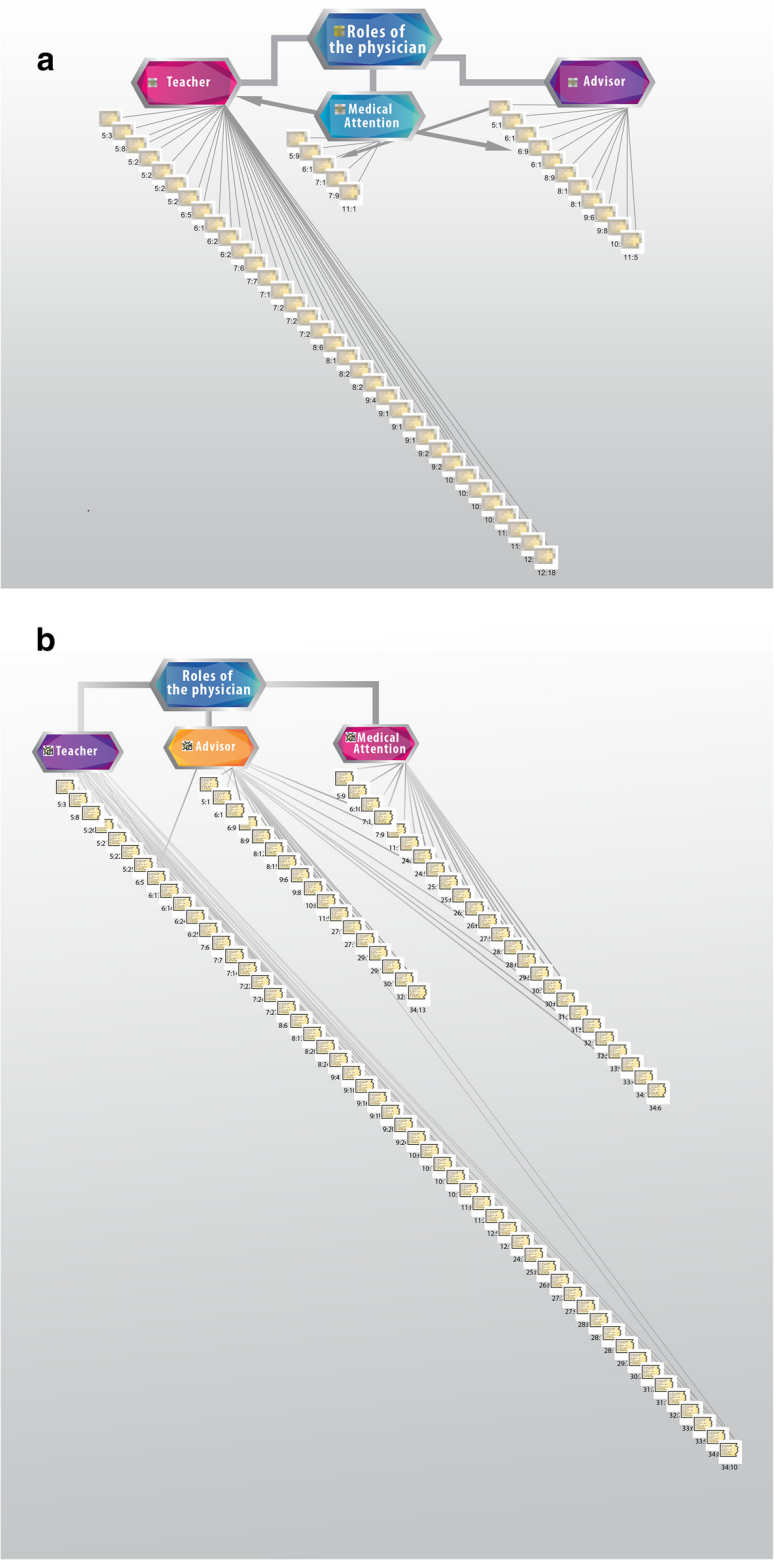
**Roles of the palliative physician.** The figure represents the roles played by healthcare personnel: counselor, educator and care provider. Values obtained with the application of the integral method of ethical discernment. In the description of dilemmas **a** and in the surveys **b**.

Advice means that a person helps another to make a decision about his or her life. Education is when the teacher helps the student to acquire a mental habit, that is, to learn something. Medical help occurs when the physician provides support to the ill person, aiding him or her to acquire the psychosomatic habit of health. The outstanding roles of the physician in palliative medicine are as educator and adviser, followed by that of provider of medical assistance. Figure 5a-b.

### Ethical discernment

The ethical deliberation in clinical practice has as a goal: the search for the good of the patient. Pellegrino and Thomasma guide us through 4 crucial steps, namely: [18]

1. Identifying the ultimate or greater good; in other words, the maximum benefit the patient searches according to his or her life options, that which makes the most sense.
2. Locating biomedical good, which refers to the good that is accomplished with medical intervention on a particular illness.
3. Taking into account the patient’s perception of his own good in a particular time and under the circumstances in which a medical decision is taken.
4. Acknowledging the good of the patient as a person capable of making decisions.

**Figure 6:**
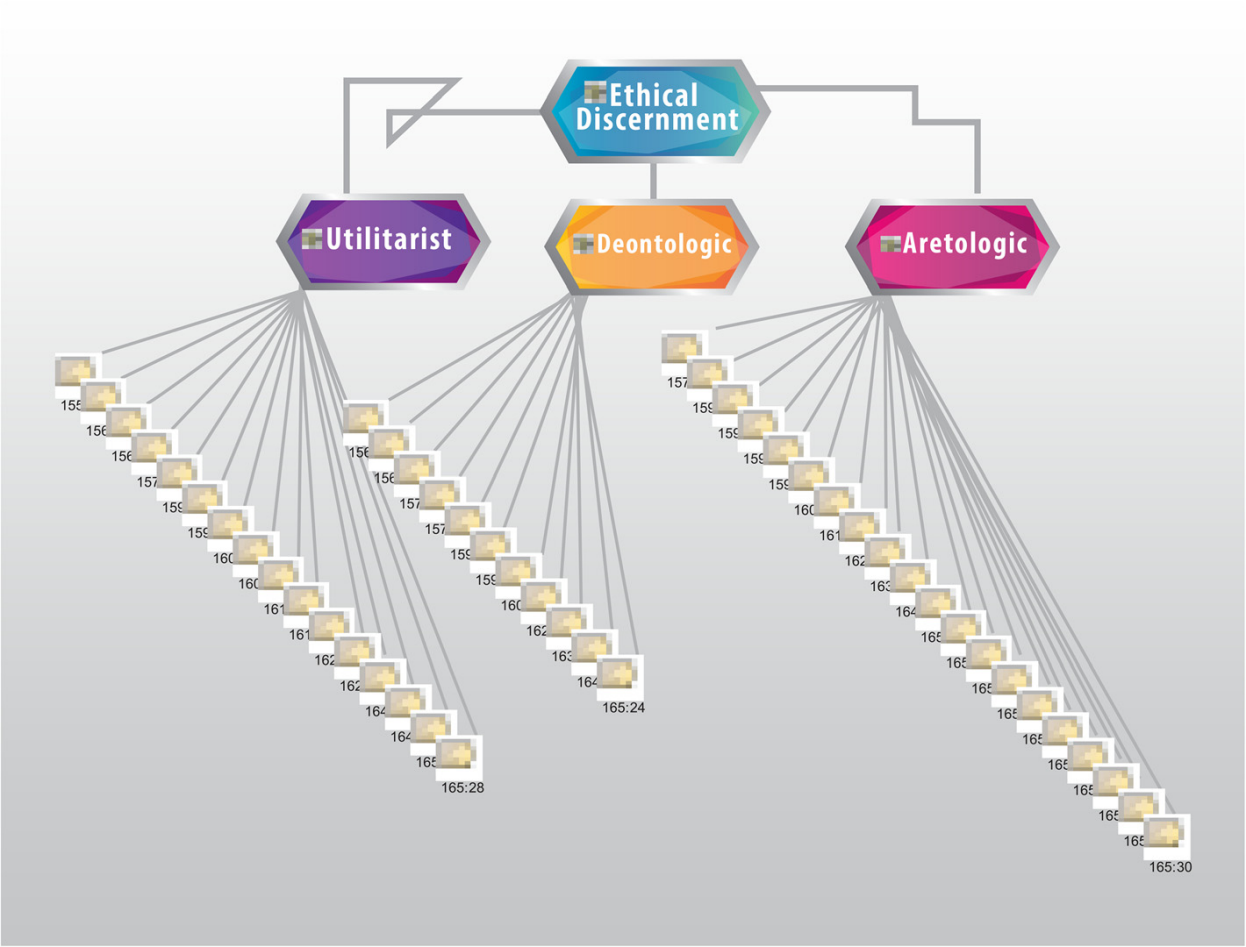
**Ethical discernment from the perspective of the main ethical theories: utilitarian, deontological and aretological.** The utilitarian evaluates the consequences of the action. Its values are efficiency, and how effective or profitable it is. Deontological theory is based on the action itself. Its values are the duties, rights and justice. The aretological vision evaluates the agent performing the action. Its main values are the person, mission and vocation. This figure shows that palliativist doctors make decisions primarily from an aretological position, secondly from a utilitarian position, and less frequently from a deontological position.

Figure 6 shows the predominance of virtue ethics, in search of the well-being of the patient, followed by the ethics of usefulness. Figure 3 shows the concurrent values that enable the search of that good, such as autonomy, justice, charity, confidentiality, compassion, trust, respect, non-maleficence, and non-discrimination.

## Discussion

Bioethics, being the center of humanities in medicine, joins the philosophy of medicine—which is the heart of the clinical practice: internal morality and the “telos” of medicine—meaning, and the goals of medicine which are to cure, to heal and to keep company. In order to achieve these purposes, healthcare providers must take into account the well-being of the patient, the role of autonomy, and the importance of the virtues of health care.

Palliative medicine is practiced especially in extreme situations, regarding life and death, suffering, pain, and multidimensional disability. Thus, the empirical study of values, virtues and roles of healthcare providers, is particularly relevant to enlighten the way in which medical decisions are being made, as well as the solving of dilemmas. This requires that providers have profound scientific knowledge, as well as synergy with the deployment of their human and professional potential.

This investigation is pioneer in unveiling the main values particular to the exercise of palliative care shown in Figures 1, 2, 3, 4, 5 and 6. Studying values in clinical practice allows us to make healthcare providers more sensible, so that they perform their duties with fuller awareness of the values and virtues they carry out, this way they will be strengthened.

The essential role of palliativists observed in this study is that of an educator. This contrasts what has been reported in other specialties such as cardiology, in which the main role is medical attention, and education plays a very role. This enlightens the duties of a palliative doctor, who takes patients in after a long treatment process.

The members of a palliative healthcare team must be predominantly educators and counselors in order to accompany patients on their last stage of life. Figure 5 shows these roles in two different sources: in the description of the dilemma (5A) and in the surveys (5B). This rapport in the results provides empirical evidence to conclude that palliative medicine is based upon certain essential values.

The next step in this research would be an analysis of patient satisfaction within these findings regarding the values recognized by palliativist physicians as part of their clinical practice. The unpacking of values in this medical branch allows fostering and strengthening them to raise physicians’ awareness of them. Our analysis considers values as a combination of principles and virtues. Virtue ethics highlights the importance of the traits of character and decision-making in ethical discernment.

Making an ethical discernment of our actions is crucial if we want our personal and professional life to develop according to the purpose of medicine. That is why it is essential to acknowledge the opportunity to revive an ethics of virtues in clinical practice. By identifying the specific virtues the palliative doctor executes on a daily basis, we will be in a better position to create educational programs ad hoc, to reinforce these values and deploy the virtues which are vital to a humanized clinical practice. This means that we must recognize the “telos” of medicine, the good of medicine as an activity; we must acknowledge and foster the essential virtues that healthcare providers must possess in order to exercise their profession successfully.

## Conclusions

By means of this study, we trust that palliativists are able to:

1. Recognize the person as the center of healthcare; to remember that the doctor-patient relationship entails an encounter between two people.
2. Promote the flourishing of the person (both the doctor and the patient) by exercising their moral power (developing use and conceptualization of their values).
3. Sensitize other health personnel to rediscover their dedication to service.
4. Create space for reflection and critical analysis of ethical dilemmas in clinical practice.
5. Promote a professional environment that is directed by academic, ethical, and social excellence.
6. Encourage the exercise of philanthropy and philotechnia in healthcare of the Mexican population and the flourishing of medical doctors.

It is worth noting that in this study we found the virtues described by Pellegrino and Thomasma [17], in actual palliative clinical practice (Figure 3). This is the first time that these virtues are identified and evaluated in palliative medicine under a qualitative analysis. Therefore, we believe that it can serve as basis for developing educational programmes and workshops aiming towards the betterment of our contemporary clinical practice. It is necessary to point out to palliative physicians the several virtues they already practice on a regular basis, and to promote greater awareness of them, in order to reinforce them and develop new ones, which will improve exercise of their profession.

By means of a rediscovery of values and virtues in palliative clinical practice, this investigation opens up new horizons for avoiding professional wearing in a medical specialty where this phenomenon is rampant. Retrieving these values and virtues will help healthcare providers to find fresh perspectives and greater motivation, giving them a new perspective of professional and personal growth.

## Endnote

^a^Axiology is a philosophical discipline which studies values and phenomena around them. Values are favourable dispositions towards desirable aims. In a Venn-Euler diagram, values constitute the universe, while virtues and principles are subsets [10].

## Abbreviations

CASC: Capacity to appraise situations and consequences
C/N: Compliance with the norm
EBM: Evidence-based medicine
VBM: Values-based medicine
